# Comparison of Two Diagnostic Assays for the Detection of Serum Neutralizing Antibody to Porcine Epidemic Diarrhea Virus

**DOI:** 10.3390/ani13040757

**Published:** 2023-02-20

**Authors:** Justin Brown, Korakrit Poonsuk, Ting-Yu Cheng, Chris Rademacher, Erin Kalkwarf, Liying Tian, Lauren A. McKeen, Chong Wang, Luis Gimenez-Lirola, David Baum, Locke A. Karriker

**Affiliations:** 1 Swine Medicine Education Center, College of Veterinary Medicine, Iowa State University, Ames, IA 50011, USA; 2 Nebraska Veterinary Diagnostic Center, University of Nebraska-Lincoln, Lincoln, NE 68588, USA; 3 Veterinary Preventive Medicine, College of Veterinary Medicine, Ohio State University, Columbus, OH 43210, USA; 4 Veterinary Diagnostic and Production Animal Medicine, College of Veterinary Medicine, Iowa State University, Ames, IA 50011, USA; 5 Department of Statistics, Iowa State University, 1121 Snedecor Hall, Ames, IA 50011, USA

**Keywords:** PEDV, neutralizing antibody, diagnostic assay

## Abstract

**Simple Summary:**

Antibodies are transmitted from a sow to her piglets through milk. Antibodies can be detected through various diagnostic assays and monitoring a sow’s antibody levels in serum may help determine if a detectable immune response could offer protection for piglets. Two diagnostic assays for the detection of neutralizing antibodies for porcine epidemic diarrhea virus were compared in this study. The assays showed moderate agreement and they can be utilized to monitor antibody levels of sows. The high-throughput neutralization assay showed advantages when compared to the fluorescent focus neutralization assay including higher specificity and greater discrimination of results.

**Abstract:**

Lactogenic immunity is important for the protection of piglets against many pathogens including porcine epidemic diarrhea virus. Circulating neutralizing antibodies levels in sow sera may help determine if a detectable immune response could confer protection to piglets. Neutralizing antibodies can be detected through various diagnostic assays. This study evaluated the diagnostic characteristics of two neutralizing antibody assays for porcine epidemic diarrhea virus neutralizing antibodies in serum of challenged gilts. Four treatment groups, control, non-vaccinated, vaccinated prior to challenge, and vaccinated following challenge, were comprised of 20 gilts. Serum sample were collected from each gilt prior to and following challenge with porcine epidemic diarrhea virus. Samples were evaluated for the presence of neutralizing antibodies via a fluorescent focus neutralization assay and a high-throughput neutralization assay. Diagnostic sensitivity and specificity for the fluorescent focus neutralization and high-throughput neutralization assays for this study were optimized at a cutoff of a dilution of 80 and 80% fluorescent reduction respectively and demonstrated moderate agreement based off the kappa statistic. The focus fluorescent neutralization and high-throughput neutralization assays can be used to monitor the status of neutralizing antibodies within animals or a population of animals. The high-throughput assay has advantages over the focus fluorescent assay in that it has a higher specificity at the indicated cut-off and the nature of the results allows for more discrimination between individual results.

## 1. Introduction

Porcine epidemic diarrhea virus (PEDV) is an *Alphacoronavirus* that has become endemic in the Americas following its emergence in the United States in 2013 [1,2,3]. Biosecurity measures are focused on keeping pathogens such as PEDV out of swine herds to mitigate the impact of disease [4]. Serology testing is a useful tool to determine if pigs have been previously exposed to and infected by a pathogen. Exposure can occur naturally (e.g., ingestion of virus-laden material) or intentionally (e.g., commingling with shedding seeder animals, oral-nasal inoculation, or gastric lavage). For the purposes of this study, exposure was achieved by intentional challenge with a PEDV positive tissue homogenate from a confirmed, clinical outbreak of PEDV. 

Song et al. [5] demonstrated serum neutralizing antibody titers did not differ between sows administered an oral or intramuscular Vero cell attenuated PEDV DR13 vaccine. This study also demonstrated that oral vaccinated sows had lower piglet mortality compared to intramuscular vaccination. Additionally, de Arriba et al. [6] demonstrated a correlation between IgA and IgG antibody-secreting cells (ASC) in the blood and gut associated lymphoid tissue. The diffuse, epitheliochorial placenta of sows prevents immune cells and antibodies from being transferred to piglets in utero making pigs agammaglobulinemic at birth [7]. Maternal immunity is transferred from sows to their piglets via mammary secretions and plays a critical role in protection against enteric pathogens such as PEDV, TGEV, and rotavirus. Therefore, piglets rely on colostrum and milk antibodies for protection from these pathogens. Serum immunoglobulins are a large contributor to colostrum whereas immunoglobulins in milk are produced within the mammary gland [8]. Thus, neutralizing antibody assays may help to determine if a detectable immune response in pre-parturient sow sera could confer protection to piglets.

The mechanisms of neutralizing antibodies include aggregation, inhibition of viral entry of the target cell, and inhibition of viral replication within the target cell [9]. Diagnostic assays have been developed to detecting neutralizing antibodies for PEDV including virus neutralization (VN), fluorescent focus neutralization (FFN), and high-throughput neutralization test (HTNT) [2,10,11,12,13]. As described by Sarmento et al. [2], the FFN assay is an improvement upon the VN assay as the assay is more sensitive because the assay is based on the detection of viral antigens expressed on the surface of infected Vero cells while the VN assay is based on the cytopathic effect caused by the virus. Additionally, FFN results can be obtained more rapidly than VN results since cytopathic effect takes longer to be observed compared to viral attachment with the FFN. The HTNT assay is further improvement upon the FFN assay as the HTNT assay relies on digital image cytometry rather than technician interpretation to obtain the results. Thus, allowing a whole plate to be read in less than 3 min, producing a quantitative measurement in fluorescent intensity, and the possibility to store images for review. Thus, the HTNT appeared to be suitable for use in commercial swine herds. 

The present study compared the diagnostic characteristics (sensitivity and specificity) and positive and negative predictive values, of two neutralizing antibody assays for PEDV, FFN and HTNT assays. This builds upon the work of Sarmento et al. [2] by testing naïve, infected, and vaccinated animals to help validate the assay for use in a high-throughput diagnostic laboratory. Based on the results of Sarmento et al. [2], our null hypothesis is that there is no difference in the diagnostic characteristics of the two assays to detect neutralizing PEDV antibodies in serum of PEDV challenged gilts. 

## 2. Materials and Methods

### 2.1. Sources and Selection of Swine

As previously described by Brown et al. [14], this study used 4 treatment groups referred to as: Control, NV (non-vaccinated), Pre (vaccinated prior to challenge), and Post (vaccinated following challenge). Each group was initially composed of 20 commercial, crossbred, gilts. A total of 60 gilts were conveniently selected from a commercial swine farm in central Iowa that had no history of PEDV. The 20 gilts in the Control group were re-used for the NV group. The NV group was comprised of only 18 pigs as two gilts were removed from the study due to complications unrelated to PEDV challenge. 

As further evidence of PEDV negative status, 12 mL of blood was collected via jugular venipuncture using a 16-gauge, 1.5-inch needle and 12 mL syringe. Serum was harvested from blood samples via centrifugation at 1.500× g for 8 min (min), split into 1 mL aliquots, and stored at −80 °C. Serum samples were tested with a wv-ELISA at the Iowa State University Veterinary Diagnostic Laboratory (ISU-VDL, Ames, IA, USA) [15]. 

### 2.2. Assignment of Animals to Treatment Groups

The sixty gilts were evenly divided into 3 groups, each comprised of 20 commercial, crossbred females. To form four treatment groups, 20 gilts were used sequentially for two of the treatment groups, CONTROL and NV. Reuse of the 20 gilts allowed for development and control of a group of PEDV positive gilts which had been exposed to PEDV and reduced the total number of animals used in the study. 

The remaining 40 gilts comprised the PRE and POST groups, 20 gilts within each group. The timeline of events by treatment group is described in Brown et al. [14]. In brief, individual blood samples were collected once weekly in each treatment group following challenge. Further elaboration is discussed in Section 2.4 and Section 2.5. The treatment group “PRE” gilts were vaccinated prior to arrival at the offsite facility. The treatment group “POST” gilts were not vaccinated until after challenge. 

### 2.3. Housing and Care of Swine

At arrival to the research facility, gilts were group housed by treatment group: all 20 gilts in a treatment group were in one pen with ad libitum feed and water. All pens were within the same room. One pen was left empty between the NV and PRE and POST groups to minimize contamination. Feed, water, room temperature highs and lows, and humidity highs and lows were monitored to ensure consistency with production standards. Health observations were conducted and recorded once daily to assess ambulation, lethargy, fecal consistency, and other clinical signs of disease. An ear tag (Integra Hog, Allflex, Dallas, TX, USA) was placed in the right ear of each gilt for individual animal identification.

### 2.4. Oral Challenge

Following a 4-day acclimation period, each gilt in CONTROL was orally exposed with PEDV using a tissue homogenate collected from a confirmed clinical outbreak of PEDV on a commercial farm. The homogenate was sequenced by the Iowa State University Veterinary Diagnostic Laboratory and found to be the US prototype strain, genogroup G2b. Ten milliliters of the homogenate were mixed with 590 mL of phosphate buffered saline and 30 mL of the mix were administered oronasally to each gilt. The inoculum was confirmed PEDV positive by real-time, reverse transcriptase polymerase chain reaction with a CT value of 19.6 and 14.23 × 10^7^ genomic copies per mL and the development of diarrhea in the gilts following challenge.

Individual blood samples were collected every 7 days following challenge via jugular venipuncture as described above through day 42 post challenge. Serum was harvested and stored as described above and samples were submitted to the ISU VDL for detection of neutralizing antibodies by a focus fluorescent neutralization assay and a high throughput virus neutralization test.

On day 60 after the initial challenge of CONTROL, the remaining 40 gilts were brought to the research facility and 18 of the CONTROL gilts became NV. Two gilts were removed from CONTROL due to conditions unrelated to the study. Following a 3-day post-arrival acclimation period, 18 gilts were re-challenged (NV) and 40 gilts (PRE and POST) were challenged, blood samples collected, serum harvested, and samples submitted for testing as described herein. 

### 2.5. Vaccination of Gilts

Forty gilts had remained at the farm of origin and were brought to the research facility 8.5 weeks after the arrival of CONTROL. Twenty gilts had been vaccinated 5 and 2 weeks prior to challenge, as recommend by the company producing the vaccine, with a commercially available, killed PEDV vaccine, genogroup G2b, (Porcine Epidemic Diarrhea Vaccine, Zoetis, Inc., Florham Park, NJ, USA) comprising the PRE group. The remaining twenty were vaccinated with the same vaccine, 1 and 3 weeks following PEDV challenge comprising the POST group. Eighteen of the original twenty CONTROL gilts remained in the study and comprised the NV group and did not receive the vaccination for PEDV.

### 2.6. Neutralizing Antibody Assays

#### 2.6.1. Fluorescent Focus Neutralization (FFN) Procedure

The FFN was performed by the Iowa State University Veterinary Diagnostic Laboratory, standard operating procedure 9.5324 [16]. Briefly, Vero 76 cells were prepared by rinsing and incubating with Trypsin-Ethylenediaminetetraacetic acid (EDTA) for 5 min at 37 °C. A 1:20 dilution of cells and cell propagation medium was made and 200 µL added to each well of a 96-well plate and incubated for 48 h. Plates were dumped and rinsed with virus inoculation medium. Serum samples were heat inactivated in a water bath at 56 °C for 30 min and then diluted in a 2-fold serial dilution starting at 1:10 in the plate. Stock virus was diluted to 100–200 foci forming units per 100 µL and 100 µL added to each well, and incubated for 60 min at 37 °C with 5% carbon dioxide (CO_2_). Test plate was dumped, rinsed and 150 µL of serum/virus mixture added to corresponding wells and incubated for 60 min at 37 °C. Serum/virus mixtures was dumped and the wells washed with virus inoculation medium. 150 µL of virus inoculation medium was added to each well and incubated for 24 h at 37 °C and 5% CO_2_. Media was removed from the plate and cells were fixed with adding 80% acetone, that has been chilled in an ice bath, for 15 min. Acetone was discarded and the plate was air dried. A 1:100 dilution of FITC conjugated PEDV NP monoclonal antibody diluted in PBS (pH 7.2) was prepared and 50 µL added to all wells and incubated at 37 °C for 60 min. The plate was washed with PBS then read using a fluorescence microscope (Olympus BX51, 10X objective, FITC filter). Positive wells do not show fluorescing plaques and negative show green plaques. Neutralizing antibody titers were expressed as the highest serum dilution with a 90% reduction of virus replication compared to the virus control. Samples with a titer ≥20 were classified as positive for PEDV neutralizing antibodies. A higher titer indicates a higher concentration of neutralizing antibody in the sample. An example of positive and negative sera samples are available in the supplementary materials. 

#### 2.6.2. High Throughput Fluorescent Neutralization Test (HTNT) Procedure

The HTNT was performed by the Iowa State University Veterinary Diagnostic Laboratory, standard operating procedure 9.5324 Version 2 [17]. Serum samples were heat inactivated in a water bath at 56 °C for 30 min. Virus stock was serial diluted 10-fold in virus inoculation medium (DMEM with 2 µg/mL Trypsin-TPCK), transferred to Vero 81 cells (ATTC^®^ CCL-81^™^), and incubated at 37 °C with 5% CO_2_ for 5 days. The last dilution in which cytopathic effect was observed was used to determine the median tissue culture infectious dose (TCID_50_) using the Spearman and Karber method [18]. 

One-hundred microliters of cell solution (Cell propagation Medium, Trypsin-EDTA, and Vero 81 cells), was diluted with 100 µL of cell propagation medium (DMEM with 10% FBS and 1% penicillin-streptomycin) to make a 1:1 dilution. A hemocytometer was used to count and calculate the dilution to make 5 × 10^4^ cell/well and added to each well of a clear bottom black 96-well plate (Coring 96 well CellBind microplate, Sigma) and incubated for 48 h at 37 °C with 5% CO_2_ to make cell confluent (100% confluent). Each plate had positive control, negative control, and virus control wells. Then, five microliters of heat-inactivated controls and samples were placed in duplicate wells to make 1:40 dilution by first 1:20 with adding 95 µL of virus inoculation medium, then 100 µL of 1:10 diluted PEDV virus (3.16 × 10^5^ TCID_50_/mL). The serum/virus mixture was incubated for 1 h at 37 °C with 5% CO_2_. All cell wells of the test plate were washed 3 times with 150 µL of cell washing medium (DMEM with 1% penicillin-streptomycin), and 150 µL of serum/virus mixture and virus controls was transferred to the test plate and incubated for 90 min at 37 °C. Serum/virus mixture was discarded and plates washed onetime with cell washing medium, as described above, followed with one time with virus inoculation medium. Virus inoculation medium (150 µL) was added to each well and incubated for 24 h at 37 °C with 5% CO_2_. Virus inoculation medium wad discarded and the plate was washed with PBS pH 7.4. Cells were fixed by adding 4 °C, 80% acetone to each well and incubated at room temperature for 15 min. Acetone was then removed and the plate was air dried for 30 min. Each well was rinsed with PBS pH 7.4. 50 µL of FITC-conjugated PEDV NP monoclonal antibody (SD6-29 clone, Medgene Labs) diluted 1:100 in PBS pH 7.4 was added to each well and incubated for 60 min at 37 °C. The plate was washed four times with PBS pH 7.4, leaving the last wash in the plate for 5 min then discarded and replaced with 100 µL of PBS pH 7.4. Plates were then read with SpectraMax i3× using SoftMax Pro 6.5 (Molecular Devices, San Jose, CA, USA) using 30 ms exposure time and 20 um focal adjustments for 541 wavelength. Samples with a Total Fluorescence Reduction (%FR) ≥85% were classified as positive for neutralizing antibodies [2]. Total fluorescence reduction was calculated as 100 − ([Average sample Total Fluorescence Intensity/Average Negative Control Total Fluorescence Intensity]) × 100). A higher total fluorescence reduction indicates higher neutralizing antibody concentrations within the samples. An example of positive and negative sera samples are available in the supplementary materials. 

### 2.7. Diagnostic Characteristics: Sensitivity and Specificity and Positive and Negative Predictive Values

Diagnostic sensitivity and specificity were calculated for the HTNT using a %FR cutoff of 70, 75, 80, 85, and 90 and the FFN using sample dilution cutoffs of 20, 40, 80, and 160 to determine the optimized cutoff. Optimized was defined as the cutoff where diagnostic specificity is maximized with minimal loss of diagnostic specificity. A subset of seventy-six known PEDV negative samples were selected from the first two sampling points for the CONTROL and POST groups. 36 known PEDV positive samples were selected from the last two sampling points for the NV group. A cutoff of greater than 85 was used to define positive samples, in congruence with the ISU VDL standard operating procedure for the assay [17]. Diagnostic sensitivity was estimated by dividing the number of known positive samples that tested positive by the total known positive samples. Diagnostic specificity was estimated by dividing the number of known negative samples that tested negative by the number of known negative samples. The cutoff that optimized diagnostic specificity and sensitivity were selected for assay comparison. For purposes of this study, diagnostic specificity (DSp) and sensitivity (DSe) were maximized, where the absolute value of the difference between sensitivity and specificity values is smallest. Positive predictive value (PPV) was estimated by dividing the number of known positive samples that tested positive (TP) by the total test positive samples (TP plus false positive [FP]). Negative predictive value (NPV) was estimated by dividing the number of known negative samples (TN) that tested negative by the total test negative samples (TN plus false negative [FN]). 

### 2.8. Statistical Analysis

There were no indeterminate results or missing data in this study. All data were analyzed using SAS Version 9.4 (SAS Instit. Cary NC). 609 observations from 60 pigs belonging to one of 4 groups were used. Two possible cutoff values (40 and 80) for the FFN assay were considered and compared to a cutoff of 85 for the HTNT. For each, a linear mixed effects model was fit to explain the effect of group on the agreement of the FFN and HTNT assays, with a pig specific random effect. All pairwise comparisons analysis of groups were performed. Further, for two of the three cutoff values used for the FFN assay, Cohen’s Kappa coefficient was calculated to determine the overall agreement of the FFN and HTNT assays. Kappa coefficients were interpreted as follows: less than 0.0, “poor” agreement; between 0.0 and 0.20, “slight” agreement; between 0.21 and 0.40, “fair” agreement; between 0.41 and 0.60, “moderate” agreement; between 0.61 and 0.80, “substantial” agreement; and between 0.81 and 1.0, “almost perfect” agreement [19]. Significance was set at *p* ≤ 0.05. Box and whisker plots were developed utilizing R software. 

## 3. Results

Table 1 shows the diagnostic sensitivity and specificity for the FFN and HTNT assays at the cutoffs selected. Diagnostic sensitivity and specificity for the two assays for this study were optimized at a cutoff of a dilution of 80 and 80% FR respectively.

**Figure 1:**
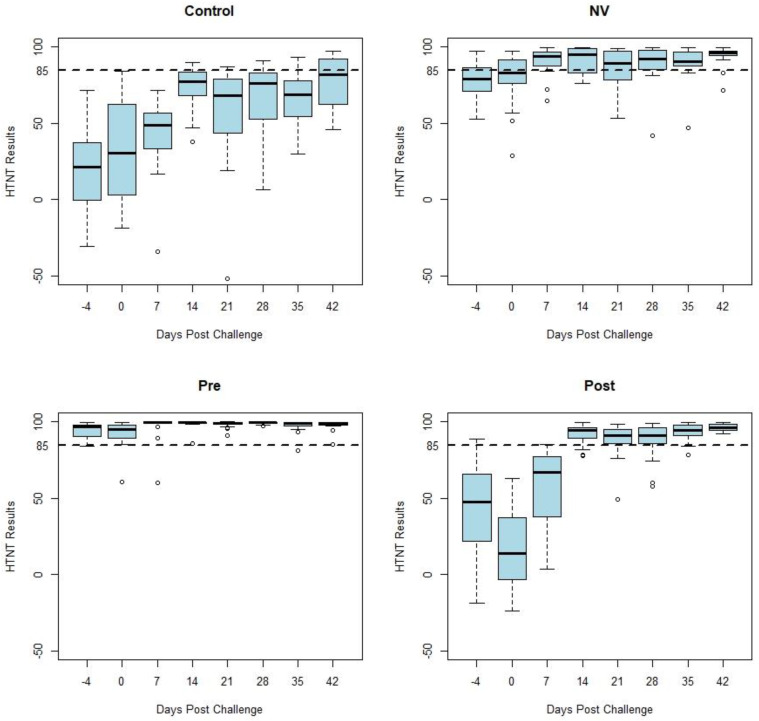
Box-and-whisker plots of the HTNT antibody response curves by the days post challenge for each treatment group. The middle bar represents the median value of the results by week. The hinges (top and bottom of the box) represent the first (Q1) and third (Q3) quartiles. The shaded region represents the interquartile range (IQR). The whiskers are determined as follows: Top whisker = Q3 + 1.5∗IQR; Bottom whisker = Q1 + 1.5∗IQR. If no data point is at the calculated value, the next data point closest to Q1 or Q3 is the limit of the whisker. Open circles (◦) indicate outliers. A cut of 85 is denoted by the dashed line at 85 on the y-axis.

Figure 1 displays box and whisker plots of HTNT results by days post challenge by study treatment group. Samples from the CONTROL and POST groups (n = 76) were classified as negative on days −4, 0, and 7 as expected due to the previous status of the pigs. The NV group developed an anamnestic response following challenge. Decreased variability occurred among the HTNT results as shown by a decrease in the range of values in both vaccinated groups (PRE and POST) following challenge with PEDV. This decreased variation can be observed in Figure 1 as the whiskers and outliers of the POST and PRE-plots become shorter 14 days post challenge.

**Figure 2:**
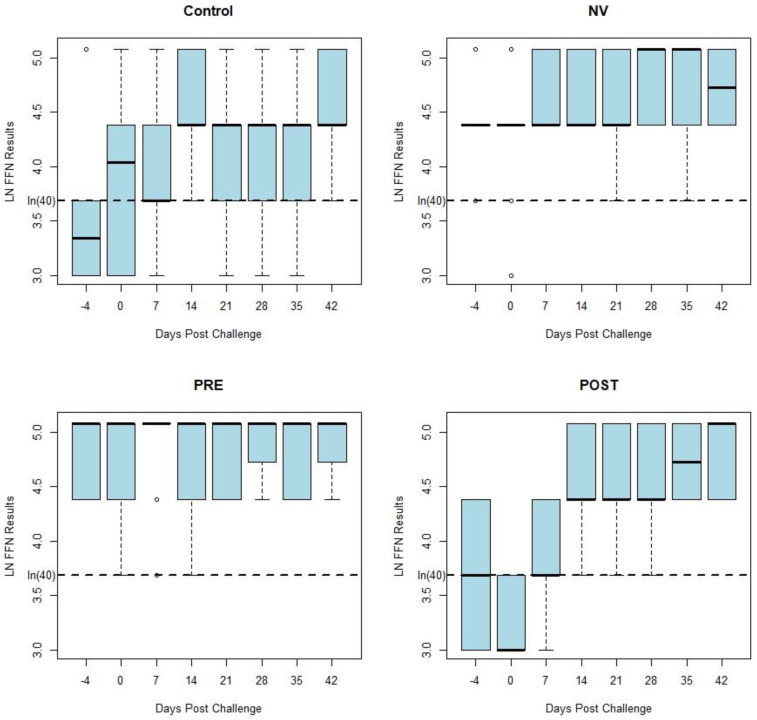
Box-and-whisker plots of the FFN antibody response curves by the days post challenge for each treatment group. The middle bar represents the median value of the results by week. The hinges (top and bottom of the box) represent the first (Q1) and third (Q3) quartiles. The shaded region represents the interquartile range (IQR). The whiskers are determined as follows: Top whisker = Q3 + 1.5∗IQR; Bottom whisker = Q1 + 1.5∗IQR. If no data point is at the calculated value, the next data point closest to Q1 or Q3 is the limit of the whisker. Open circles (◦) indicate outliers. A cut of 40 is denoted by the dashed line at ln(40) on the y-axis.

Figure 2 displays box and whisker plots of natural log transformed FFN results by days post challenge by treatment group, which have similar results as the HTNT assay. Both the HTNT (Figure 1) and FFN (Figure 2) results show a response to challenge and vaccination as seen with an increase in %FR and titers from both assays. 

While similar in magnitude, the variation of each assay’s results was reduced following homologous challenge (NV). Decreased variation also occurred following vaccination (PRE and POST). This variation of neutralizing antibody values by week post exposure decreases following vaccination and homologous challenge with PEDV (Figure 1 and Figure 2). There was a similar tendency in the decrease of variation of neutralizing antibody seen in both assays’ results of vaccinated and homologous challenged treatment groups. Homologous challenge of PEDV and second vaccinations produced an anamnestic response generating a higher level of neutralizing antibodies with reduced variation between pigs compared to pre-challenge and post-vaccination values as measured by both assays (Figure 1).

Based on analysis of the diagnostic sensitivity and specificity, the cutoffs of 80 (titer) and 85 (%FR) were selected to analyze test agreement for the FFN and HTNT assays respectively. The kappa test demonstrated “moderate” agreement of 0.5257 with a 95% confidence interval of 0.461, 0.5904 of the assays for the cutoff 80 (titer) and 85 (%FR) for the FFN and HTNT assays. 

## 4. Discussion

The present study compared the PEDV antibody response curves of two neutralizing antibody assay platforms, the FFN and HTNT using samples collected after exposure of pigs with PEDV and vaccination as described previously [14]. Reduction in the range of the assay results is evidence that the population of gilts included in this study produced a uniform neutralizing antibody response following exposure or vaccination. A uniform immune response within a population is critical to the prevention of PEDV. Irregular responses in the population will allow susceptible animals to become infected and disease to continue to spread throughout the population. The decrease in the range of the assay results is most obvious in the HTNT results compared to the FFN results. This decrease in variation might be explained by the nature of each test’s results; the HTNT results are ratios of continuous data that can range from −100 to 100 whereas the FFN results are categorical by dilution (i.e., 1:20, 1:40, 1:80 etc.). The anamnestic response produced by a second exposure to PEDV generated a more homogenous distribution and reduced variation of antibody concentrations in the gilts used in this study. This homogeneity of antibody concentrations may correspond to improved protection against subsequent infections. Additionally, a higher number of non-specific reactors, ie. false positive results, were identified by the FFN assay compared to the HTNT. 

We hypothesize that reduced variation and an anamnestic response are associated with homogenous herd immunity and protection from disease, even though clinical signs were still observed in the PRE group as described previously [14].

The clinical impact and severity of disease in these animals was not evaluated for the purposes of this study. Disease manifests differently between adults and neonates with more severe clinical signs and death in neonates compared to adult animals [1,20]. While circulating neutralizing antibody levels alone do not confer protection [21], they may be used to assess an antibody response following intentional exposure or vaccination administration in herds. While absence of viremia is characteristic of PEDV, viremia has been reported in piglets up to 4-weeks of age [22,23]. Thus, circulating neutralizing antibody may influence the clinical course of disease [21]. However, lactogenic immunity, specifically IgA in mammary secretions would be a more accurate measurement of protection for piglets [21]. 

The assays described in this study could be used to monitor serum neutralizing antibody following vaccination administration in groups of sows or gilts in addition to collecting mammary secretions to test for lactogenic/secretory IgA. It would be beneficial if the data generated from these assays could predict the quantity and stability of lactogenic immunity produced following vaccination. However, that was not analyzed in the present study. Serum IgG antibody responses following vaccination have been shown to be predictive of colostral IgG response, but there was no correlation between serum neutralizing antibodies and those of mammary secretions [15]. While there was no correlation between serum neutralizing antibodies and those of mammary secretions, vaccinated animals were shown to have higher neutralizing antibodies in mammary secretions compared to control animals [15]. Therefore, as supported by, Bjustrom-Kraft, et al. [15], if serum neutralizing antibodies are high, then IgG and IgA in mammary secretions may also be increased. Thus, collecting serum samples prior to movement of the females to the farrowing house would minimize stress and pain induced during periparturient collection of blood and mammary secretions that may negatively impact the sow and litter [24].

**Figure 3:**
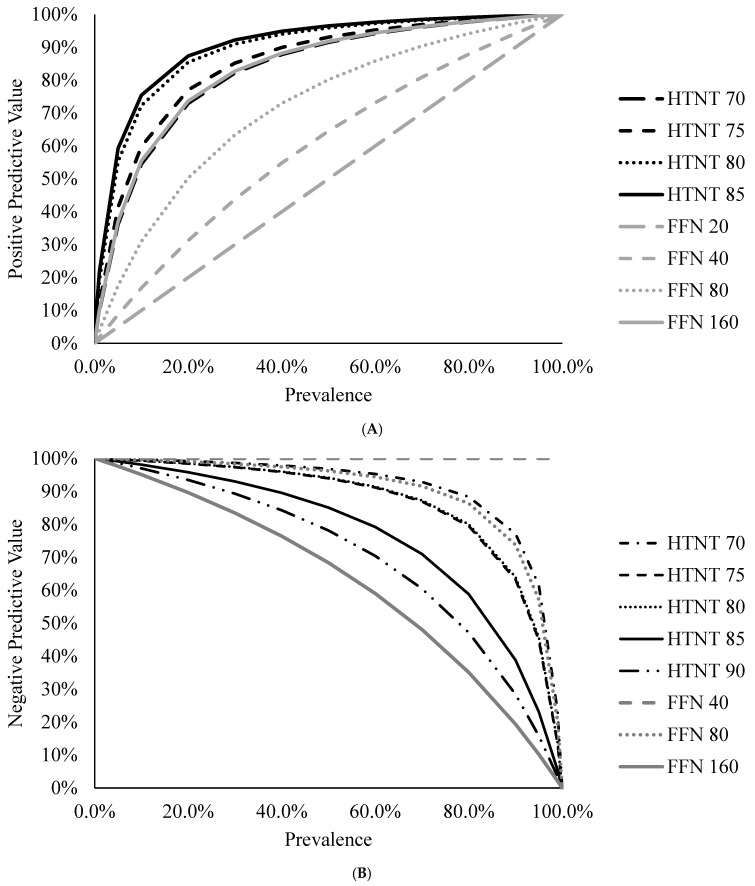
(**A**). Positive Predictive values for the HTNT (black lines) and FFN (gray lines) assays at various cutoffs. A cutoff of 90 is not represented for the HTNT. (**B**). Negative predictive values or the HTNT (black lines) and FFN (gray lines) assays at various cutoffs. A cutoff of 20 is not represented for the FFN assay.

It is important for users to understand how the cut-off value can affect the diagnostic sensitivity and specificity of an assay. Increasing the cut-off value will decrease the number of false positive results and increase the number of false negative results. Inversely, decreasing the cut-off value will increase the number of false positive results and decrease the number of false negative results. This knowledge allows a user to choose which cut-off value to use for their specific situation. For this study, based on results of this study and previous results published by Sarmento et al. [2], we chose 80 %FR for the HTNT and dilution of 80 for FFN. At these cut-offs diagnostic specificity is higher for the HTNT (97%) compared to the FFN (76%) and provides higher positive predictive value from the HTNT compared to the FFN. It must also be understood that predictive values are dependent on the prevalence of disease [25,26]. In this study, based off the classified samples the prevalence was 32.1% (36 positive and 76 negative samples). As prevalence increases, the PPV would also increase because the tested animal (sample) is more likely to be positive; as prevalence decreases, the NPV would decrease. Figure 3A,B illustrate this for the HTNT and FFN assays at the various cutoffs evaluated in this study following methods described by Erb [25]. Therefore, when considering diagnostic results, one should consider the diagnostic sensitivity and specificity of the assay and understand the prevalence within the group if using predictive values. 

The HTNT has superior diagnostic sensitivity and specificity compared to the FFN. When analyzing antibody results, this is ideal to minimize the number of false positive and false negative results. For example, when using a cutoff of 80 for the FFN, we observed at sensitivity of 97% and specificity of 76%. This means that there would be few false negative results but there would be a large number of false positives. If results are falsely categorized as positive, then this may lead to a misinterpretation that a herd has antibodies to PEDV when in fact they do not. Since the HTNT has higher diagnostic sensitivities and specificities across various cutoffs, it is the preferable assay for the detection of PEDV antibodies. Results from Sarmento et al. [2] showed much higher diagnostic specificities for the FFN and comparable sensitivities and specificities for the HTNT. These diagnostic assays showed moderate agreement according to the kappa statistic (κ). One weakness of κ is that one test must be considered the gold standard. When comparing two imperfect assays, this can skew the interpretation of κ. For this study we evaluated the diagnostic performance of the assays in conjunction with κ to determine that the test agree but may have different utility. 

An additional advantage of the HTNT over the FFN assay is that the HTNT results are calculated from continuous data compared to the categorical values (titrations) from the FFN. The continuous nature of the HTNT data allows for greater discrimination of sample results in order to describe the neutralizing antibody status of sows. As reported by Sarmento et al. [2], the HTNT has a shorter read-time of a 96 well plate to less than 4 min allowing for a faster turnaround on results. 

Circulating systemic antibodies may contribute to piglet protection against PEDV infections [21]. However, there is no literature on what quantity of circulating neutralizing antibody in the sow would confer PEDV protection to piglets. High levels of circulating antibody lead to higher concentrations in the colostrum and afford increased protection. Further evaluation is warranted to determine an association between, when during gestation to collect the samples, and colostral antibody protection against PEDV infection. Lactogenic and mucosal immunity is of vital importance for the survivability of pigs exposed to PEDV. Antibodies collect in the milk via the gut-MG-sIGA axis and are passed to the piglets to confer protection. Langel et al. [27] found that gilts exposed during the second trimester had significantly higher levels of circulating IgA, IgG, and neutralizing antibodies compared to first and third trimester gilts. Additionally, Langel et al. [27] found that piglets of gilts exposed to PEDV in the second trimester had 100% survival rate compared to less than 87.2% in other trimesters suggesting that high serum IgA, IgG, and neutralizing antibodies are associated with piglet survival in the face of PEDV. Although serum antibodies do not confer protection for PEDV, results from our study suggest serum neutralizing antibody levels are associated with protection against PEDV infection. 

As previously reported by Brown et al. [14], PEDV is shed in the feces following homologous challenge and vaccination of gilts. The present study utilized samples from the same animals as Brown et al. [14]. further shows that increased serum neutralizing antibody following challenge exposure or vaccination does not influence the shedding pattern of the virus as antibody levels increased following challenge and vaccination. Further studies are warranted to confirm if the amount of virus shed in the feces is associated with the quantity of neutralizing antibody that is present in the serum [21]. 

## 5. Conclusions

The null hypothesis of this study was that there was no difference in the diagnostic characteristics of two PEDV neutralizing antibody assays, FFN and HTNT. Results show that these assays moderately agree based on the kappa statistic while there is variation in their diagnostic characteristics and predictive values based on the cutoff for the assay. These assays could be used to measure a vaccination response of a herd. Failure to reject the null hypothesis shows that HTNT is the preferred test for the detection of PEDV neutralizing antibodies in serum. 

Following vaccination in both the PRE and POST groups, this study found a majority of results as positive. In the PRE group, decreased variation among the results also followed challenge. These results suggest swine practitioners could measure serum neutralizing antibody in order to determine if PEDV vaccines are being administered properly in the herd.

## Figures and Tables

**Table 1:** Diagnostic sensitivity (DSe), Diagnostic specificity (DSp), Positive Predictive Value (PPV) and Negative Predictive Value (NPV) for HTNT and FFN assays using various cutoffs listed in the second row of the table. For example, for the HTNT assay at a cutoff of 70, the DSe = 0.97 (97%), the DSp = 0.91 (91%), PPV = 0.83 (83%) and NPV = 0.95 (95%). U = undefined.

	HTNT Selected %FR Cutoff					FFN Selected Sample Dilution Cutoff			
	70	75	80	85	90	20	40	80	160
DSe	0.97	0.94	0.94	0.83	0.72	1.00	1.00	0.97	0.56
DSp	0.91	0.93	0.96	0.97	1.00	0.00	0.45	0.76	0.95
PPV	0.83	0.87	0.92	0.94	1.00	0.32	0.46	0.66	0.83
NPV	0.95	0.97	0.97	0.93	0.88	U	1.00	0.98	0.82

## Data Availability

The datasets used and analyzed during the current study are available from the corresponding author on reasonable request.

## Supplementary Materials

The following supporting information can be downloaded at: https://www.mdpi.com/article/10.3390/ani13040757/s1, Figure S1: negative and positive assay results.
