# Supplementary material for: Comparison of Two Diagnostic Assays for the Detection of Serum Neutralizing Antibody to Porcine Epidemic Diarrhea Virus
Source: Animals (Basel). 2023 Feb 20;13(4):757. doi: 10.3390/ani13040757 (PMC9951927; doi:10.3390/ani13040757)

Figure S1: Negative and Positive Assay Results

No virus neutralization (Negative) | Virus neutralization (Positive)

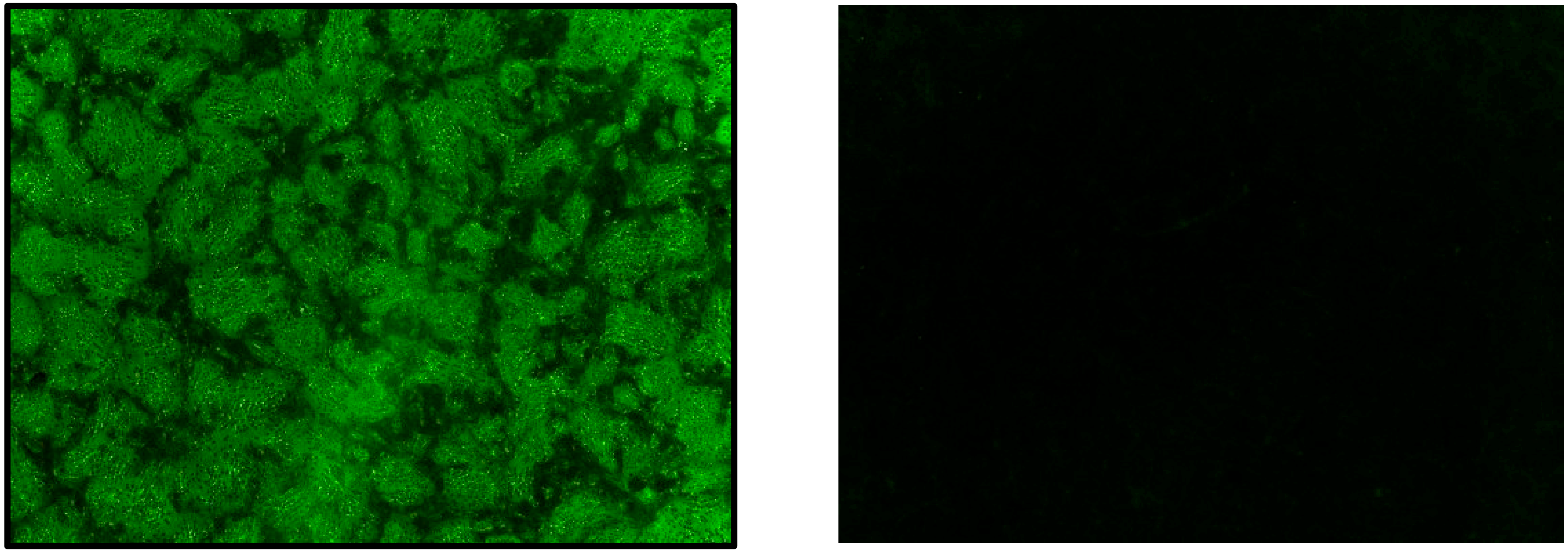

Supplement: Supplementary file 1 [file animals-13-00757-s001.zip › animals-2165808-supplementary.pdf]
